# Supplementary material for: Impact of sleep on the microbiome of oral biofilms
Source: PLoS One. 2021 Dec 9;16(12):e0259850. doi: 10.1371/journal.pone.0259850 (PMC8659294; doi:10.1371/journal.pone.0259850)
Supplement: S1 Table — Abbreviations: BM, buccal mucosa; HP, hard palate; GM, gingival mucosa; SUB, subgingival dental biofilm; SUP, supragingival dental biofilm; SV, saliva; TD, tongue dorsum; Post, post-sleeping schedule; Pre, pre-sleeping schedule. (DOCX) [file pone.0259850.s001.docx]

|  |  | Actinobacteria | Bacteroidetes | Firmicutes | Fusobacteria | Proteobacteria | other |
| --- | --- | --- | --- | --- | --- | --- | --- |
| BM | Post | 6.13% | 18.05% | 50.71% | 4.97% | 16.47% | 3.67% |
|  | Pre | 6.50% | 11.00% | 60.89% | 4.95% | 15.56% | 1.07% |
| HP | Post | 11.36% | 20.16% | 52.53% | 4.02% | 9.88% | 2.03% |
|  | Pre | 11.12% | 11.37% | 58.86% | 4.85% | 12.94% | 0.84% |
| GM | Post | 1.20% | 22.40% | 54.28% | 7.04% | 13.48% | 1.56% |
|  | Pre | 0.33% | 24.40% | 55.11% | 7.45% | 11.38% | 1.30% |
| SUB | Post | 18.91% | 28.76% | 16.29% | 10.26% | 19.00% | 6.74% |
|  | Pre | 13.27% | 30.28% | 14.66% | 14.52% | 21.65% | 5.65% |
| SUP | Post | 29.07% | 21.49% | 19.27% | 6.74% | 18.38% | 5.05% |
|  | Pre | 15.84% | 23.07% | 23.49% | 7.25% | 26.92% | 3.45% |
| SV | Post | 6.28% | 31.07% | 29.64% | 7.32% | 18.58% | 7.07% |
|  | Pre | 8.52% | 22.01% | 31.96% | 7.00% | 25.23% | 5.26% |
| TD | Post | 17.21% | 27.91% | 27.46% | 9.30% | 14.17% | 3.95% |
|  | Pre | 19.14% | 18.62% | 30.25% | 11.13% | 18.62% | 2.23% |

**Table S1 Relative abundance of bacterial taxa in oral biofilms at phylum level.**

Abbreviations are shown below; BM: buccal mucosa, HP: hard palate, GM: gingival mucosa, SUB: subgingival dental biofilm, SUP: supragingival dental biofilm, SV: saliva, TD: tongue dorsum, Post: post-sleeping schedule, Pre: pre-sleeping schedule.
